# Predictors of Short-Term Mortality in Patients with Ischemic Stroke

**DOI:** 10.3390/medicina59061142

**Published:** 2023-06-13

**Authors:** Silvina Iluţ, Ştefan Cristian Vesa, Vitalie Văcăraș, Dafin-Fior Mureșanu

**Affiliations:** 1 Department of Neurosciences, “Iuliu Haţieganu” University of Medicine and Pharmacy, 8 Victor Babeş Street, 400012 Cluj-Napoca, Romania; silvina.ilut@yahoo.com (S.I.); vvacaras@umfcluj.ro (V.V.); dafinm@ssnn.ro (D.-F.M.); 2 Department of Pharmacology, Toxicology and Clinical Pharmacology, “Iuliu Haţieganu” University of Medicine and Pharmacy, 23 Gheorghe Marinescu Street, 400337 Cluj-Napoca, Romania; 3 RoNeuro Institute for Neurological Research and Diagnostic, 37 Mircea Eliade Street, 400364 Cluj-Napoca, Romania

**Keywords:** short-term mortality, ischemic stroke, predictors

## Abstract

*Background and Objectives*: The purpose of this study is to investigate the predictive factors for intrahospital mortality in ischemic stroke patients. We will examine the association between a range of clinical and demographic factors and intrahospital mortality, including age, sex, comorbidities, laboratory values, and medication use. *Materials and Methods*: This retrospective, longitudinal, analytic, observational cohort study included 243 patients over 18 years old with a new ischemic stroke diagnosis who were hospitalized in Cluj-Napoca Emergency County Hospital. Data collected included the patient demographics, baseline characteristics at hospital admission, medication use, carotid artery Doppler ultrasound, as well as cardiology exam, and intrahospital death. *Results*: Multivariate logistic regression was used to determine which variables were independently associated with intrahospital death. An NIHSS score > 9 (OR—17.4; *p* < 0.001) and a lesion volume > 22.3 mL (OR—5.8; *p* = 0.003) were found to be associated with the highest risk of death. In contrast antiplatelet treatment (OR—0.349; *p* = 0.04) was associated with lower mortality rates. *Conclusions*: Our study identified a high NIHSS score and large lesion volume as independent risk factors for intrahospital mortality in ischemic stroke patients. Antiplatelet therapy was associated with lower mortality rates. Further studies are needed to explore the potential mechanisms underlying these associations and to develop targeted interventions to improve patient outcomes.

## 1. Introduction

Ischemic stroke is a leading cause of morbidity and mortality worldwide, accounting for approximately 87% of all stroke cases [1]. Despite advances in acute stroke management, including thrombolysis and endovascular therapy, the overall mortality rate of ischemic stroke remains high, particularly in patients with severe strokes [2]. Identifying factors that predict intrahospital mortality in ischemic stroke patients is essential for providing timely and appropriate treatment and improving patient outcomes.

Several studies have investigated the predictors of intrahospital mortality in ischemic stroke patients. Age, comorbidities, and stroke subtype have been identified as important predictors of mortality [3,4,5]. Additionally, some studies have suggested that certain laboratory values, such as elevated blood glucose and leukocytosis, may be predictive of intrahospital mortality [6,7]. Other potential predictors include hypertension, hyperlipidemia, smoking status, and prior stroke history [8].

However, the results of these studies have not been consistent, and some potential predictors have not been well studied. For example, while hypertension is a known risk factor for stroke, its association with intrahospital mortality in ischemic stroke patients is unclear. Moreover, the impact of other factors, such as race/ethnicity and sex, on intrahospital mortality has not been well characterized [9].

The purpose of this study is to investigate the predictive factors for intrahospital mortality in ischemic stroke patients. We will examine the association between a range of clinical and demographic factors and intrahospital mortality, including age, sex, comorbidities, laboratory values, and medication use.

## 2. Materials and Methods

### 2.1. Patients

This retrospective, longitudinal, analytic, observational cohort study included 243 patients over 18 years old with a new ischemic stroke diagnosis who were hospitalized in Cluj-Napoca Emergency County Hospital (CNECH), the second largest tertiary stroke center in Romania, between 1 January 2022 and 31 August 2022 due to ischemic stroke. Patients were identified through electronic charts based on the relevant International Classification of Diseases, Tenth Revision (ICD-10) codes for stroke. A stroke diagnosis was given according to the Guidelines for the Early Management of Patients with Acute Ischemic Stroke: 2019 Update to the 2018 Guidelines for the Early Management of Acute Ischemic Stroke: A Guideline for Healthcare Professionals from the American Heart Association/American Stroke Association.

The hospital had a catchment area of around 683,018 habitants/year in 2022. Most ischemic stroke cases in this area are in contact with our hospital at the initial admission as part of acute management and follow up. Thus, it is possible to estimate population-based rates for stroke. Population figures for the incidence rates were obtained from the Romanian National Institute of Statistics [10].

The following ICD-10 codes were searched for: I63.3, I63.4 and I63.5. All clinical and neuroradiological assessments for the identified ischemic stroke cases were reassessed and confirmed by a senior neurologist. Patients with repeated presentations due to chronic ischemic stroke during the study period were counted as one case in the analysis. Demographic, clinical, radiological and potential risk factors were investigated for all patients.

The Medical Ethics Committee of the Neurology Hospital approved the study.

### 2.2. Data Collection

The data collected included the patient demographics (age, sex, address, and medical history), baseline characteristics at hospital admission (physical examination, neurological examination, laboratory results (e.g., vascular or coagulation disorders)), medication use, carotid artery Doppler ultrasound, as well as cardiology exam, and intrahospital death. The National Institutes of Health Stroke Scale (NIHSS) score was calculated for all patients. We diagnosed stroke at admission using computed tomography (CT). The initial lesion volume was noted. Lesion volume was calculated using the formula for ellipsoid volume V = 4/3 π × (A/2) × (B/2) × (C/2), where A is the greatest lesion diameter in the axial plane, B is the lesion diameter at 90° to A in the axial plane, and C is the craniocaudal diameter of the lesion.

### 2.3. Statistical Analyses

Statistical analysis was performed using MedCalc^®^ Statistical Software version 20.218 (MedCalc Software Ltd., Ostend, Belgium). Qualitative data were presented as absolute and relative values. The normality of the distribution for quantitative data was assessed using the Shapiro–Wilk test and was expressed as the mean and standard deviation. Comparisons between groups were performed using Student’s *t*-test or chi-square tests, whenever appropriate. ROC analysis was used to establish a cutoff value for the association of age with stroke events. Variables that achieved statistical significance in the univariate analysis were used for the multivariate logistic regression. Multivariate logistic regression was used to identify variables that were independently associated with stroke. Statistical significance was considered at *p* < 0.05.

## 3. Results

There were 32 patients who died during hospitalization and 211 who survived. The median hospitalization time was 7 (5; 10) days. Comparisons between deceased and surviving patients (Table 1) showed that age, the presence of AF, mitral insufficiency, antiplatelet treatment, and hemorrhagic transformation were associated with intrahospital death. We calculated a cutoff of 79 years, above which the probability of intrahospital death increased (AUC = 0.686; 95% CI 0.623 to 0.744; *p* < 0.001). We calculated for NIHSS a cutoff of 9, above which the probability of intrahospital death increased (AUC = 0.887; 95% CI 0.840 to 0.924; *p* < 0.001). We calculated for lesion volume a cutoff of 22.3 mL, above which the probability of intrahospital death increased (AUC = 0.828; 95% CI 0.774 to 0.873; *p* < 0.001). Patients with a larger lesion volume were more likely to undergo hemorrhagic transformation (33.8 vs. 14.2 mL; *p* = 0.001).

Multivariate logistic regression was used to determine which variables were independently associated with intrahospital death (Table 2). A high NIHSS score and a large lesion volume were found to have the highest risk of death. In contrast, antiplatelet treatment was associated with lower mortality rates.

## 4. Discussion

The purpose of this study was to investigate the predictive factors for intrahospital mortality in ischemic stroke patients. Our findings suggest that a high NIHSS score and a large lesion volume were independently associated with an increased risk of intrahospital death. In contrast, antiplatelet treatment was associated with lower mortality rates. Advanced age and the presence of AF were predictive of early mortality only in univariate analysis. However, in multivariate analysis, their statistical threshold was slightly exceeded. The significant association between advanced age and intrahospital mortality in ischemic stroke patients found in our study is in line with previous research findings. Even though in multivariate analysis, the statistical value was slightly over 0.05 (0.1), the trend is clear. The studies by Furlan et al. [11] and Schmidt et al. [12] also identified age as an independent predictor of in-hospital mortality in stroke patients, consistent with our results. Advanced age has been identified as a significant risk factor for stroke, with increasing age associated with a higher risk of stroke incidence and severity [13,14,15]. This is likely due to the presence of multiple comorbidities and a higher burden of vascular risk factors in elderly patients. Moreover, the elderly population often has reduced physiological reserves and may be more susceptible to complications, such as infections and thromboembolic events, which can contribute to increased mortality risk [7]. These complications can also be exacerbated by the presence of other comorbidities such as diabetes, hypertension, and cardiovascular disease. Therefore, advanced age in ischemic stroke patients should be considered a high-risk factor for intrahospital mortality, and clinicians should take measures to minimize potential complications in these patients. Preventive measures, such as aggressive control of vascular risk factors, including hypertension, diabetes, and hyperlipidemia, have been shown to reduce the incidence and mortality of ischemic stroke [16]. Furthermore, the early identification and treatment of acute complications, such as infections, have been shown to improve outcomes in elderly stroke patients [7]. Timely initiation of rehabilitation programs is also crucial for functional recovery in elderly patients. Therefore, interventions aimed at minimizing complications and promoting functional recovery should be implemented in elderly stroke patients to reduce intrahospital mortality risk.

Our study found that the presence of AF was a predictor of intrahospital mortality in ischemic stroke patients only in univariate analysis, although in multivariate analysis, the p value was close the statistical significance threshold and showed a clear trend. Previous studies have reported an association between AF and worse outcomes in stroke patients [17,18,19]. AF is a common cardiac arrhythmia that affects a significant proportion of stroke patients and is associated with a higher risk of stroke and worse stroke outcomes [20]. The pathophysiology underlying the association between AF and poor stroke outcomes is multifactorial, but it is believed to be related to an increased risk of embolic stroke due to the formation of thrombi in the atria and subsequent embolization to the brain [21]. Wańkowicz et al. showed that patients with AF that took statins prior the stroke had a better outcome [22]. In our study, we did not find that statins improved survival.

Our findings revealed a significant link between a high NIHSS score (>9) and an increased risk of early mortality in patients with acute ischemic stroke. This observation is consistent with several previous studies that reported similar results. Firstly, our findings align with the study conducted by Adams et al. which demonstrated that a higher NIHSS score at admission was associated with a greater likelihood of early mortality in stroke patients [23]. Ramachandran showed recently that NIHSS is a valid prognosticator of the aftermath in patients with ischemic stroke [24]. Smith et al. found a strong correlation between a high NIHSS score and increased mortality rates within the first 30 days after stroke onset [25]. Additionally, our results are in line with the research conducted by Kortazar-Zubizarreta et al., who conducted a similar study in a single center and reported that a high NIHSS score was one of the main independent predictors of early mortality in ischemic stroke patients [26]. These findings were further supported by the study conducted by Moraes et al., which showed that a high NIHSS score at admission was significantly associated with early mortality in both ischemic and hemorrhagic stroke patients [27]. Furthermore, our study contributes to the existing body of literature by demonstrating the prognostic value of the NIHSS score in predicting early mortality in stroke patients. The level of NIHSS associated with early mortality ranged between 4 and 14, depending on the study.

Our study also identified an association between antiplatelet treatment and lower mortality rates in ischemic stroke patients. This finding is supported by previous studies that demonstrated the effectiveness of antiplatelet therapy in reducing the risk of recurrent stroke [28]. Antiplatelet therapy, including aspirin and clopidogrel, is commonly used in the secondary prevention of stroke, and our findings suggest that it may also have a beneficial effect on intrahospital mortality in ischemic stroke patients. The mechanisms underlying the association between antiplatelet therapy and reduced mortality in ischemic stroke patients are not fully understood, but it is believed to be related to the prevention of secondary vascular events and the improvement of cerebral blood flow [29]. Furthermore, antiplatelet therapy may have anti-inflammatory and anti-thrombotic effects, which could reduce the risk of complications and improve outcomes [30]. Antiplatelet medications can potentially increase the risk of bleeding, including hemorrhagic transformation [31]. The decision to use antiplatelet therapy in patients with a high risk for hemorrhagic transformation or with hemorrhagic transformation is complex and is made on an individual basis. The potential benefits and risks of antiplatelet treatment need to be carefully considered, taking into account factors such as the severity of bleeding, the overall clinical condition of the patient, and the risk of recurrent ischemic stroke.

Our results provide evidence for a significant association between a large lesion volume and increased early in-hospital mortality in stroke. Previous studies have shown that larger lesion volumes are associated with more severe neurological deficits, delayed recovery, and poorer functional outcomes [32,33]. Additionally, stroke patients with larger lesion volumes exhibit a higher likelihood of developing complications, such as hemorrhagic transformation, and increased intracranial pressure [34]. The impact of lesion volume on early mortality has been reported across different stroke subtypes. In ischemic stroke, a larger infarct size has been consistently associated with increased mortality rates [33,35]. Similarly, in hemorrhagic stroke, larger hematoma volumes have been linked to higher mortality rates due to increased mass effect and surrounding tissue damage [36].

## 5. Conclusions

Our study identified a high NIHSS score and a large lesion volume as independent risk factors for intrahospital mortality in ischemic stroke patients. Antiplatelet therapy was associated with lower mortality rates. Advanced age and the presence of AF could be predictor factors for early mortality. Further studies are needed to explore the potential mechanisms underlying these associations and to develop targeted interventions to improve patient outcomes.

## Figures and Tables

**Table 1:** Comparison between deceased and survivors.

Variables		Deceased (n = 32)	Survivors (n = 211)	*p*
Age		76.5 ± 10.5	68.5 ± 12.3	<0.001
Sex	F	13 (40.6%)	78 (37%)	0.8
	M	19 (59.4%)	133 (63%)	
Environment	Urban	14 (43.8%)	107 (50.7%)	0.5
	Rural	18 (56.3%)	104 (49.3%)	
Smoker	No	31 (96.9%)	190 (90.0%)	0.3
	Yes	1 (3.1%)	21 (10.0%)	
NIHSS score		18 (14; 20)	7 (5; 12)	<0.001
Arterial hypertension	No	3 (9.4%)	36 (17.1%)	0.398
	Yes	29 (90.6%)	175 (82.9%)	
Atrial Fibrillation (AF)	No	13 (40.6%)	156 (73.9%)	<0.001
	Yes	19 (59.4%)	55 (26.1%)	
Congestive heart failure	No	26 (81.3%)	193 (91.5%)	0.103
	Yes	6 (18.8%)	18 (8.5%)	
Cardiomyopathy	No	31 (96.9%)	203 (96.2%)	1
	Yes	1 (3.1%)	8 (3.8%)	
Myocardial infarction	No	31 (96.9%)	193 (91.5%)	0.482
	Yes	1 (3.1%)	18 (8.5%)	
Ischemic cardiomyopathy	No	23 (71.9%)	162 (76.8%)	0.701
	Yes	9 (28.1%)	49 (23.2%)	
Diabetes mellitus type II	No	28 (87.5%)	155 (73.5%)	0.135
	Yes	4 (12.5%)	56 (26.5%)	
Peripheral obliterative arteriopathy	No	31 (96.9%)	200 (94.8%)	1
	Yes	1 (3.1%)	11 (5.2%)	
Dyslipidemia	No	27 (84.4%)	157 (74.4%)	0.273
	Yes	5 (15.6%)	54 (25.6%)	
Aortic stenosis	No	30 (93.8%)	201 (95.3%)	0.662
	Yes	2 (6.3%)	10 (4.7%)	
Aortic insufficiency	No	32 (100%)	196 (92.9%)	0.231
	Yes	0 (0.0%)	15 (7.1%)	
Atheromatous plaque	No	32 (100%)	195 (92.4%)	0.140
	Yes	0 (0.0%)	16 (7.6%)	
Thrombolysis	No	29 (90.6%)	161 (76.3%)	0.110
	Yes	3 (9.4%)	50 (23.7%)	
Thrombectomy	No	31 (96.9%)	196 (92.9%)	0.702
	Yes	1 (3.1%)	15 (7.1%)	
Anticoagulant therapy	No	16 (50%)	117 (55.5%)	0.699
	Yes	16 (50%)	94 (44.5%)	
Antiplatelet therapy	No	21 (65.6%)	75 (35.5%)	0.001
	Yes	10 (34.4%)	137 (64.5%)	
Statin therapy	No	23 (71.9%)	148 (70.1%)	1
	Yes	9 (28.1%)	63 (29.9%)	
Lesion volume (mL)		37.9 (24.9; 46.4)	14.2 (8.6; 22.3)	<0.001
Hemorrhagic transformation	No	23 (71.9%)	187 (88.6%)	0.02
	Yes	9 (28.1%)	24 (11.4%)	

**Table 2:** Multivariate logistic regression.

	B	*p*	OR	95% C.I. for OR	
				Lower	Upper
Age > 79 years	0.855	0.103	2.352	0.840	6.585
NIHSS score > 9	2.859	<0.001	17.441	3.631	83.768
Lesion volume > 22.3 mL	1.773	0.003	5.886	1.842	18.809
AF	0.774	0.130	2.167	0.797	5.896
Antiplatelet therapy	−1.054	0.045	0.349	0.124	0.979
Hemorrhagic transformation	0.791	0.206	2.206	0.648	7.512
Constant	−2.152	<0.001	0.116		

## Data Availability

Data available on request due to privacy or ethical issues. The data presented in this study are available on request from the corresponding author.
